# P-81. Clinical Characteristics and Outcomes of Salmonella Osteomyelitis: A Systematic Review and Meta-Analysis of Individual Cases

**DOI:** 10.1093/ofid/ofaf695.310

**Published:** 2026-01-11

**Authors:** Ashley Ungor, Molly Courtright, Paddy Ssentongo, Affan Faisal, Meredith A Schade, Talha Riaz

**Affiliations:** College of Medicine, University of Arizona, Tucson; College of Medicine, University of Arizona, Tucson; Penn State Health Milton S. Hershey Medical Center, Hershey, PA; King Edward Medical University, Lahore, Punjab, Pakistan; Penn State MS Hershey Medical Center, hummelstown, Pennsylvania; University of Arizona College of Medicine Tucson, Tucson, Arizona

## Abstract

**Background:**

Salmonella osteomyelitis is a rare but serious extraintestinal manifestation of invasive Salmonella infection. Characterization of its clinical presentation, risk factors, imaging findings, and treatment strategies remains limited, particularly in distinguishing vertebral from non-vertebral cases.

**Fig 1:**
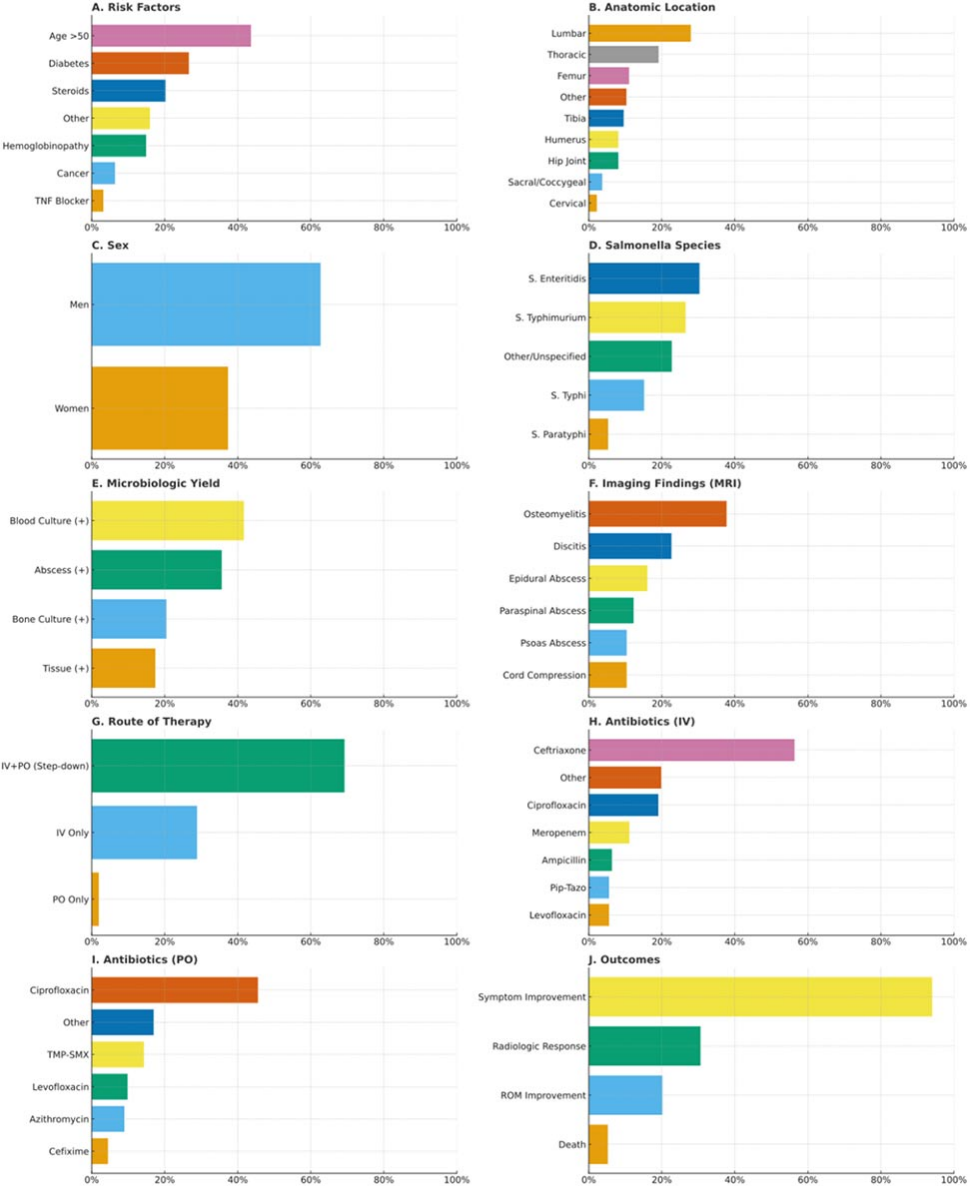
Clinical characteristics, microbiologic findings, imaging features, treatment approaches, and outcomes among patients with Salmonella osteomyelitis. Panel A shows patient risk factors; Panel B, anatomic locations of infection; Panel C, sex distribution; Panel D, Salmonella species identified; Panel E, microbiologic yield; Panel F, magnetic resonance imaging findings; Panel G, routes of antibiotic therapy; Panel H, intravenous antibiotic agents; Panel I, oral antibiotic agents; and Panel J, clinical outcomes. Percentages are shown based on the available number of cases for each domain.

**Methods:**

We systematically searched the Scopus, PubMed and Embase databases for case reports and case series describing Salmonella osteomyelitis in adults, including both vertebral and non-vertebral infections, published through 1994-2024. From eligible articles, we extracted demographics and risk factors, clinical manifestations, microbiologic data, imaging findings, antimicrobial regimens (intravenous, oral, and step-down therapy), and patient outcomes. Data were summarized using descriptive statistics.

**Results:**

Among 136 patients, the median age was 45 years, and 62.7% were men (Fig. 1). Vertebral involvement was observed in 53% (72/136), commonly in the lumbar (28%) and thoracic (19%) regions. Non-vertebral infections affected the femur (11%), tibia (10%), and hip joint (8%). The most common symptoms were localized pain (84%) and fever (74%). Risk factors included age >50 years (44%), diabetes (27%), corticosteroid use (20%), and hemoglobinopathies (15%). Blood cultures were positive in 59%, while bone (93%) and abscess (94%) cultures were highly positive. Non-typhoidal Salmonella (S. Enteritidis and S. Typhimurium) was most common, with MRI revealing osteomyelitis (38%), discitis (23%), and abscesses (28%). Most patients (69%) received a combination of intravenous and oral antibiotics, with ceftriaxone and fluoroquinolones being most common. Surgical intervention was required in 73%. Among 134 patients with outcome data, 7 (5.2%) died.

**Conclusion:**

Salmonella osteomyelitis, though uncommon, often presents with vertebral involvement and requires medical and surgical management. Clinicians should maintain a high index of suspicion of osteoarticular Salmonellosis among patients with diabetes, advanced age and immunosuppression. Prompt imaging, microbiologic diagnosis, and combination of antimicrobial and surgical intervention is essential to optimize outcomes and reduce morbidity.

**Disclosures:**

All Authors: No reported disclosures

